# Neutrophile-to-Lymphocyte Ratio (NLR) Identifies Patients with Coronavirus Infectious Disease 2019 (COVID-19) at High Risk for Deterioration and Mortality—A Retrospective, Monocentric Cohort Study

**DOI:** 10.3390/diagnostics12051109

**Published:** 2022-04-28

**Authors:** Jutta Rose, Flurina Suter, Eva Furrer, Ataman Sendoel, Melina Stüssi-Helbling, Lars C. Huber

**Affiliations:** 1 Department of Internal Medicine, Clinic for Internal Medicine, City Hospital Zurich Triemli, 8063 Zurich, Switzerland; melina.stuessi-helbling@stadtspital.ch (M.S.-H.); lars.huber@stadtspital.ch (L.C.H.); 2 Faculty of Medicine, University of Zurich (UZH), 8006 Zurich, Switzerland; ataman.sendoel@uzh.ch; 3 Institute for Regenerative Medicine, University of Zurich (UZH), 8952 Schlieren, Switzerland; 4 Master Program in Biostatistics, University of Zurich (UZH), 8006 Zurich, Switzerland; flurina.suter2@uzh.ch; 5 Department Biostatistics, Institute for Epidemiology, Biostatistics and Prevention, University of Zurich (UZH), 8006 Zurich, Switzerland; eva.furrer@uzh.ch

**Keywords:** COVID-19, SARS-CoV-2, neutrophile-to-lymphocyte ratio, NLR, disease severity, mortality

## Abstract

Among people infected with SARS-CoV-2, the determination of clinical features associated with poor outcome is essential to identify those at high risk of deterioration. Here, we aimed to investigate clinical phenotypes of patients hospitalized due to COVID-19 and to examine the predictive value of the neutrophil-to-lymphocyte ratio (NLR) in a representative patient collective of the Swiss population. We conducted a retrospective monocentriccohort study with patients hospitalized due to COVID-19 between 27 February and 31 December 2020. Data were analyzed descriptively, using the binary logistic regression model, proportional odds logistic regression model, competing risk analysis, and summary measure analysis. A total of 454 patients were included in our study. Dyspnea, elevated respiratory rate, low oxygen saturation at baseline, age, and presence of multiple comorbidities were associated with a more severe course of the disease. A high NLR at baseline was significantly associated with disease severity, unfavorable outcome, and mortality. In non-survivors, NLR further increased during hospital stay, whereas in survivors, NLR decreased. In conclusion, our data emphasize the importance of accurate history taking and clinical examination upon admission and confirm the role of baseline NLR as a surrogate marker for increased disease severity, unfavorable outcome, and mortality in patients hospitalized due to infection with SARS-CoV-2.

## 1. Introduction

Severe acute respiratory syndrome coronavirus type 2 (SARS-CoV-2) has spread globally, causing recurrent pandemic waves of coronavirus infectious disease 2019 (COVID-19) in almost all countries. The clinical spectrum of SARS-CoV-2 infection is broad and ranges from asymptomatic infection, mild upper respiratory tract illness to severe viral pneumonia with respiratory failure and death. Hence, the determination of clinical and laboratory markers associated with poor outcome is essential to identify low-risk patients that can be managed as outpatients and those at high risk for deterioration. Patients at high risk of deterioration should be considered for close monitoring and early transfer to the intensive care unit (ICU). As such, initial severity assessment is a key part of guiding management and treatment escalation.

Several signs, symptoms, and risk factors are associated with poor outcome. Among these, shortness of breath, chest pain, low oxygen saturation at baseline, and risk factors such as age, male gender, and presence of multiple comorbidities have been reported to be associated with poor clinical outcome and high risk for mortality [1,2,3]. 

The white blood cell ratio, in particular, the neutrophil-to-lymphocyte ratio (NLR), is considered a surrogate marker of systemic hyperinflammation and an independent predictor of poor outcome associated with COVID-19 [4]. In fact, baseline NLR not only accurately identifies patients at high risk for clinical deterioration, but also discriminates high versus low mortality risk in patients with COVID-19 [4,5]. In terms of prognostic significance, the NLR has been shown to be superior compared to the neutrophil granulocytes and lymphocytes alone [4,5,6].

Moreover, the platelet-to-lymphocyte ratio (PLR) has emerged as a risk stratification tool for patients infected with SARS-CoV-2 [7]. A large meta-analysis investigating the predictive role of PLR in patients hospitalized due to COVID-19 showed that higher levels of PLR at time of admission are associated with increased morbidity and mortality [7].

Both markers, however, are limited by the lack of specificity and are also found elevated in other chronic conditions such as hematological disorders, cardiovascular and neurodegenerative disease, and malignancy [8,9,10,11,12].

To our knowledge, little is known about clinical phenotypes and the role of clinical predictors for patients infected with SARS-CoV-2 in Switzerland [4,13]. In the present study, we aimed to investigate clinical phenotypes of patients hospitalized due to COVID-19 and to examine the predictive value of NLR, PLR, neutrophil granulocytes, lymphocytes, platelets, and C-reactive protein (CRP) in a representative patient collective of the Swiss population. 

## 2. Materials and Methods

### 2.1. Study Population and Data Collection

We conducted a retrospective, monocentric cohort study at City Hospital Zurich Triemli, Switzerland. Consenting adult patients with COVID-19 that were admitted to our hospital between 27 February and 31 December 2020 were included in our study. Patients were excluded if they were hospitalized not due to COVID-19 or if laboratory values were lacking. Moreover, patients transferred from other hospitals and those pretreated for more than two days prior to hospital admission were excluded. Finally, we excluded all patients that were already enrolled in a clinical trial. Informed consent was collected from the majority of patients with COVID-19. The cantonal ethics committee Zurich granted a surrogate permission for those patients, for whom no consent could be obtained (BASEC-Nr. 2020-01852, 18 August 2020). The study was conducted in accordance with the declaration of Helsinki.

Data were collected from electronic medical records using a standardized collection form from the day of admission (baseline data) and during the course of hospital stay. Data were randomly double-checked by two independent physicians. Data collection included baseline demographics (age, gender, comorbidities) and clinical characteristics including symptoms, vital signs, laboratory markers (NLR, PLR, neutrophil granulocytes, lymphocytes, platelets, CRP), and outcome (non-invasive ventilation (NIV), mechanical ventilation, need for extra corporeal membrane oxygenation (ECMO), and death). Details are provided in Table S1. Blood cell count was measured with DxH 800 of Beckman Coulter or ADVIA 2120 of Siemens, and CRP was measured with Roche Cobas C501. The number of measurements varied among the patients.

### 2.2. Definitions

Every patient with a positive test for COVID-19 taken from respiratory specimens by real-time reverse-transcription polymerase chain-reaction (RT-PCR) assay (CDC ncov-2019 rT PCR) was considered a confirmed case, as recommended by the Center for Disease Control and Prevention, Atlanta. Disease severity refers to the worst severity level reached during the hospital stay and was defined according to the World Health Organization (WHO) classification [14]. On the basis of disease severity, the patients were divided into non-severe (mild and moderate disease) and severe (severe and critical disease) groups. An unfavorable outcome was defined as a composite outcome of need for non-invasive, mechanical ventilation, extracorporeal membrane oxygenation, or death. Mortality was defined as all-cause in-hospital mortality. Definitions of baseline characteristics and comorbidities are provided in Table S1. For longitudinal NLR measurements, days −3 to 0 were defined as days before therapy initiation. Days 1 to 7 were defined as days under therapy. ‘Therapy’ refers to all medication established during the course of hospital stay, including antibiotics and off-label therapies such as hydroxychloroquine, remdesivir, and dexamethasone.

### 2.3. Statistical Analysis

Continuous variables were expressed as mean +/− standard deviation (SD), median, interquartile range (IQR), and absolute range. Analysis of variance (ANOVA) was used to compare means between different groups. Categorical variables were presented as percentages and analyzed by the chi-squared test. A p-value less than 0.05 was considered significant. 

NLR, PLR, neutrophil granulocytes, lymphocytes, and platelets were log-transformed to ensure a better overview of widely spread values. Proportional odds logistic regression models were used to describe the association between disease severity (ordinal variable) and laboratory results. Binary logistic regression models were used to illustrate the effect of laboratory results on unfavorable outcome and mortality. Multivariable models were adjusted for clinically relevant variables such as age, gender, and comorbidities (arterial hypertension, diabetes mellitus, obesity). Separate calculation of the effect size from every single comorbidity was not possible due to limited sample number. As such, a variable was created when at least one of the comorbidities was present.

Longitudinal log (NLR) measurements were visualized using a trajectory plot. Each patient’s data were displayed as an individual curve (indicated as a grey line). Day 0 was defined as the mean of all measurements before therapy initiation. Values between day 1 to 7 were defined as days under therapy. Here, individual measured values were used. A summary measure analysis was used to estimate an intercept and the slope for each patient’s data. On the basis of outcome, patient’s data were divided into survivors and non-survivors. Finally, the median intercept and slope obtained for each group (survivors vs. non-survivors) were calculated. All statistical analyses were performed using the R programming language (R Core team, 2020, R: A Language and Environment for Statistical Computing. R Foundation for Statistical Computing, Vienna, Austria.).

## 3. Results

### 3.1. Cohort

**Figure 1:**
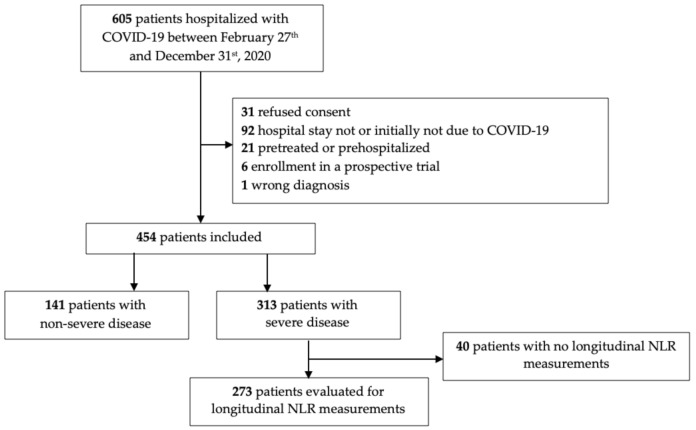
Cohort included in the study.

A total of 605 patients with laboratory-confirmed SARS-CoV-2 infection were treated at City Hospital Zurich Triemli Switzerland between 27 February and 31 December 2020. In total, 454 patients were included in our study. Of the patients, 313 (68.9%) were classified as severe cases, and 141 (31.1%) as non-severe cases. A total of 273 (87.2%) of the severe cases were evaluated for longitudinal NLR measurement. Details are provided in Figure 1. 

### 3.2. Demographic and Clinical Phenotypes of COVID-19 Patients

Of the 454 patients, 141 (31.1%) were classified as non-severe and 313 (68.9%) as severe cases. The average ages of the non-severe and severe groups were 61 years (min 18, max 95) and 69 years (min 22, max 96), *p* = 0.001, respectively. The proportion of men was higher in the severe group (66.5%) than in the non-severe group (58.9%). The median time from onset of symptoms to hospital admission was 6 days (IQR, 3 to 9 days) in the non-severe and 7 days (IQR, 4 to 10 days) in the severe group (*p* = 0.028). At the time of hospital admission, clinical features such as dyspnea (34.0% vs. 53.4%, *p* < 0.001), elevated respiratory rate (20 (IQR, 16 to 24) vs. 24 (IQR, 20 to 28), *p* < 0.001), and low oxygen saturation (96 (IQR, 94 to 98) vs. 91 (IQR, 88 to 94), *p* < 0.001) were related to an increased risk for disease progression.

Patients classified as severe cases had more underlying comorbidities than non-severe cases (no comorbidities 38.3% vs. 23.0%; ≥3 comorbidities 14.2% vs. 28.1%, *p* = 0.001, respectively). In detail, arterial hypertension, diabetes mellitus, malignant disease, and obesity were associated with an increased disease severity (41.8 vs. 53.0%, *p* = 0.035; 17.0 vs. 30.4%, *p* = 0.004; 6.4 vs. 14.4%, *p* = 0.023; 14.9 vs. 26.2%, *p* = 0.011, respectively).

Of the patients included in the cohort, 411 (90.5%) had been discharged and 43 died (mortality rate 9.5%). A total of 92 cases (20.3%) met the definition of unfavorable outcome. There were no deaths within the non-severe cases, while the mortality rate within the severe cases was 13.7%. Details are provided in Table 1.

### 3.3. Association of Laboratory Results with Disease Severity, Unfavorable Outcome, and Mortality

NLR at hospital admission was documented for 454 patients. The median NLR was 5.3 (IQR 3.3 to 8.5). The median NLR was higher in severe cases than in non-severe cases (IQR 6 vs. 3.5, *p* < 0.001). The same was observed for PLR and CRP (242.4 vs. 194.6, *p* = 0.002; 84.8 vs. 31.6, *p* < 0.001).) Details are provided in Table 1.

Moreover, as shown in Table 2, median NLR, PLR, and CRP were significantly higher in non-survivors than among survivors (8.2 vs. 5, *p* < 0.001; 268.3 vs. 215.5, *p* = 0.008, 102 vs. 60.8, 0.001). 

Multivariable analysis adjusted for clinically relevant variables (age, sex, and comorbidities such as arterial hypertension, diabetes mellitus, and obesity) demonstrated that a high log (NLR) was strongly associated with disease severity (OR 2.56, 95% CI 1.97–3.32, *p* < 0.0001), unfavorable outcome (OR 2.04, 95% CI 1.46–2.91, *p* < 0.0001), and mortality (OR 1.82, 95% CI 1.14–2.95, *p* = 0.013). An increase in log (neutrophil granulocytes) was significantly associated with disease severity and unfavorable outcome (OR 3.52, 95% CI 2.48–5.00, *p* < 0.0001; OR 3.65, 95% CI 2.2–6.26, *p* < 0.0001, respectively) but not with mortality. A decrease in log (lymphocytes) was significantly associated with disease severity (OR 0.63, 95% CI 0.45–0.88, *p* = 0.006) but not with unfavorable outcome or mortality. 

Of interest, neither levels of CRP nor levels of PLR were associated with disease severity (OR 1.01, 95%, CI 1.01–1.01 *p* < 0.0001; OR 1.26, 95% CI 0.95–1.68, *p* = 0.11, respectively), unfavorable outcome (OR 1.01, 95% CI 1.01–1.01, *p* < 0.0001; OR 1.16, 95% CI 0.79–1.72, *p* = 0.46, respectively), or mortality (OR 1, 95% CI 1–1.01, *p* = 0.042; OR 1.37, 95% CI 0.79–2.46, *p* = 0.27, respectively). 

Of great importance, most of these effects were affected by age, gender, and the presence of comorbidities, showing significant association with disease severity, unfavorable outcome, and mortality. Details are provided in Table 3.

### 3.4. Longitudinal NLR Measurements

**Figure 2:**
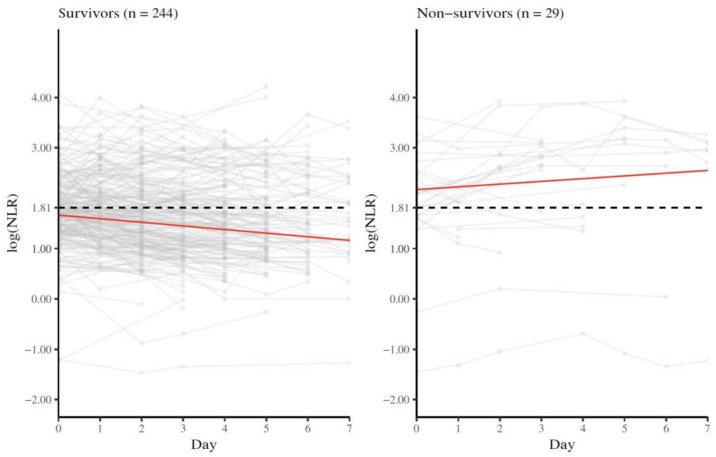
Trajectory plot of NLR measurements in survivors and non-survivors: n = number of patients. Each grey line indicates an individual patient. The median intercept and the slope of each group obtained from the summary measure analysis are displayed in red. The black, dashed line indicates the cut-off value of log (1.81), corresponding to a NLR of 6.11, as suggested by Cai et al. [15].

A total of 273 patients with severe disease were evaluated for longitudinal NLR measurement. In total, 244 (89.4%) patients survived and 29 (10.6%) died. The median intercept of log (NLR) was <1.81 in survivors and >1.81 in non-survivors. This threshold of log (NLR) 1.81 (equivalent to NLR 6.11) was chosen as suggested by Cai and coworkers, showing that a NLR > 6.11 was associated with an increased risk for mortality [15]. The median slope of log (NLR) revealed a reduction of −0.07 per day in survivors and an increase of 0.05 per day in non-survivors. Trajectory plots are shown in Figure 2. Individual slopes are shown in Figure S1.

## 4. Discussion

In this retrospective analysis, we investigated clinical phenotypes and the predictive value of NLR in patients admitted to a Swiss tertiary hospital with laboratory confirmed SARS-CoV-2 infection. 

Here, we could show that dyspnea, increased respiratory rate, and low oxygen saturation upon admission are associated with increased disease severity. These findings suggest that patients at high risk for deterioration already present in poor condition upon admission. One explanation might be the longer time interval between symptom onset and hospitalization. This is supported by Han et al., who claim that early admission to hospital might prevent disease progression [16]. The presence of dyspnea is a well-known marker for in-hospital mortality and resource use [17]. Our data confirm these findings in the cohort of patients with COVID-19. In addition, we found that an increased respiratory rate is associated with the risk for clinical deterioration. However, respiratory rate is often not recorded or, worse, not even measured in the hospital setting [18]. Our results provide an important reminder for careful history taking and clinical examination, particularly to regularly assess respiratory rate.

We also found that a high baseline NLR is strongly associated with increased disease severity, unfavorable outcome, and—in contrast to the levels of neutrophilic granulocytes and lymphocytes alone—mortality. In fact, a NLR > 6.11 at baseline seems to be associated with high risk of mortality [15]. This is along the line with other studies, confirming that NLR accurately identifies patients at high risk for clinical deterioration. Moreover, NLR appears to discriminate high versus low mortality risk in patients with COVID-19 [4,5]. However, as confirmed by our data provided here, these effects are strongly affected by age, gender, and comorbidities [8,9,10,11,12]. Of note, not only NLR at admission but also longitudinal NLR measurements might adjust the risk for mortality during hospitalization. As such, NLR might provide a feasible and cost-effective tool to detect patients at high risk for deterioration at hospital admission and during the further course of hospitalization in resource-limited settings.

Along the line of the published literature, we could confirm that baseline PLR is significantly elevated in severe cases of COVID-19 and among non-survivors [7]. However, as recently reported, we found no association between PLR and disease severity, unfavorable outcome, or mortality [19]. Our data also emphasize that PLR is not suitable as an early prognostic marker to predict clinical deterioration or in-hospital mortality in patients with COVID-19.

Of note, thrombocytopenia seems to be a hallmark of severe COVID-19 infections with higher mortality and a more severe course of COVID-19 disease, which of course also affects to predictive value of the PLR, at least relatively [20,21]. The exact mechanisms by which NLR and PLR differ in their prognostic role as discriminators for the outcome of patients with COVID-19 are yet to be unraveled. As shown by Lin and coworkers, both hematological markers are subjected to different extent by genetic and environmental factors, including BMI and smoking status, whereas the association between inflammatory markers (e.g., CRP and interleukin-6) was low [22]. The limited prognostic yield of PLR has also been reported in patients undergoing extracorporeal membrane oxygenation due to respiratory failure and cardiogenic shock [23]. Consistent with these and other findings, the NLR seems to be superior to the PLR in terms of predicting disease severity and mortality in patients with COVID-19 [24].

Our study has several limitations. The main limitations of this study are the singlecenter design, the small sample size, and the fact that data collection was not performed prospectively. As such, p-values have to be interpreted with caution. Another limitation is that we only performed adjustment for age, gender, arterial hypertension, diabetes mellitus, and obesity. Moreover, NLR and PLR are known to be affected by concomitant bacterial infections, administration of antibiotics, COVID-19-specific therapy, and the presence of additional chronic diseases [8,9,10,11,12]. The association between laboratory markers and outcome has to be considered with caution. 

Our study also has considerable strengths: to our knowledge, this is the first study investigating the predictive role of NLR in COVID-19 in a Swiss cohort. In contrast to previous studies, we excluded patients that were not hospitalized due to COVID-19 and those with prior hospitalization that were already upon COVID-19-specific therapy to minimize the effects of confounding. Moreover, we have followed the current WHO classification for disease severity allowing standardizing of outcome data.

## 5. Conclusions

In conclusion, our data highlight the importance of accurate history taking and clinical examination upon admission and confirm the role of baseline NLR, compared to other inflammation markers, as a surrogate marker for increased disease severity, unfavorable outcome, and mortality. Importantly, repetitive NLR measurements might predict mortality in patients hospitalized due to infection with SARS-CoV-2.

## Figures and Tables

**Table 1:** Clinical characteristics of patients hospitalized due to COVID-19 (n = 454).

Variable	Overall	Non-Severe	Severe	*p*-Value	NA (%)
**Demographic Data**					
n (%)	454	141 (31.1)	313 (68.9)		
Gender				0.146	0.0
Male = yes (%)	291 (64.1)	83 (58.9)	208 (66.5)		
Female = yes (%)	163 (35.9)	58 (41.1)	105 (33.5)		
Age (median (min, max))	68 (18, 96)	61 (18, 95)	69 (22, 96)	0.001	0.0
Age distribution				<0.001	0.0
18–44 years (%)	47 (10.4)	30 (21.3)	17 (5.4)		
45–64 years (%)	156 (34.4)	46 (32.6)	110 (35.1)		
65–79 years (%)	155 (34.1)	40 (28.4)	115 (36.7)		
≥80 years (%)	96 (21.1)	25 (17.7)	71 (22.7)		
**Symptoms**					
Time between onset of symptoms and hospitalization (days) (median IQR)	7 (4, 10)	6 (3, 9)	7 (4, 10)	0.028	5.1
Cough (%)	292 (64.3)	90 (63.8)	202 (64.5)	0.968	0.0
Fever (%)	253 (55.7)	81 (57.4)	172 (55.0)	0.694	0.0
Headache (%)	63 (13.9)	24 (17.0)	39 (12.5)	0.248	0.0
Chest pain (%)	64 (14.1)	20 (14.2)	44 (14.1)	1.000	0.0
Dyspnea (%)	215 (47.4)	48 (34.0)	167 (53.4)	<0.001	0.0
Myalgia, arthralgia, malaise (%)	264 (58.1)	83 (58.9)	181 (57.8)	0.917	0.0
Nasal congestion (%)	12 (2.6)	8 (5.7)	4 (1.3)	0.017	0.0
Gastrointestinal symptoms (%)	91 (20.0)	27 (19.1)	64 (20.4)	0.847	0.0
Sore throat (%)	28 (6.2)	10 (7.1)	18 (5.8)	0.735	0.0
Anosmia (%)	13 (2.9)	4 (2.8)	9 (2.9)	1.000	0.0
**Vital signs**					
Systolic blood pressure (mmHg) (median (IQR))	138 (123, 153)	133 (122, 147)	139 (124, 155)	0.080	2.4
Diastolic blood pressure (mmHg) (median (IQR))	76 (66, 84)	77 (67, 84)	76 (65, 84)	0.462	2.4
Heart rate (/min.) (median (IQR))	88 (77, 100)	86 (76, 94)	88 (77, 102)	0.077	1.8
Oxygen saturation (%) without supplemental oxygen (median (IQR))	93 (89, 96)	96 (94, 98)	91 (88, 94)	<0.001	1.8
Respiratory rate (/min.) (median (IQR))	23 (18, 28)	20 (16, 24)	24 (20, 28)	<0.001	11.7
Temperature (°C) (median (IQR))	37.7 (37.0, 38.4)	37.6 (36.9, 38.3)	37.8 (37.1, 38.4)	0.123	5.1
GCS < 15 (%)	33 (7.3)	8 (5.7)	25 (8.0)	0.494	0.0
**Laboratory results**					
NLR (median (IQR))	5.3 (3.3, 8.5)	3.5 (2.2, 6.5)	6.0 (3.9, 9.7)	<0.001	1.3
PLR (median IQR))	222.9 (152.3, 334.3)	194.6 (139.1, 283.0)	242.4 (156.8, 356.6)	0.002	1.3
Neutrophile granulocytes (10^9^ cells/l) (median (IQR))	4.5 (3.0, 6.0)	3.6 (2.4, 5.0)	4.8 (3.7, 6.5)	<0.001	1.3
Lymphocytes (10^9^ cells/l) (median (IQR))	0.9 (0.6, 1.2)	1.0 (0.7, 1.3)	0.8 (0.6, 1.1)	<0.001	1.3
Platelets (10^9^ cells/l) (median (IQR))	185.0 (145.5, 240.8)	186.5 (148.8, 230.3)	184.5 (141.8, 244.3)	0.680	0.4
CRP (mg/L) (median (IQR))	65.3 (29.2, 120.0)	31.6 (12.7, 72.3)	84.8 (45.5, 137.0)	<0.001	0.7
**Comorbidities**					
Number of comorbidities				0.001	0.0
0 (%)	126 (27.8)	54 (38.3)	72 (23.0)		
1 (%)	120 (26.4)	40 (28.4)	80 (25.6)		
2 (%)	100 (22.0)	27 (19.1)	73 (23.3)		
≥3 (%)	108 (23.8)	20 (14.2)	88 (28.1)		
Arterial hypertension (%)	225 (49.6)	59 (41.8)	166 (53.0)	0.035	0.0
Diabetes mellitus (%)	119 (26.2)	24 (17.0)	95 (30.4)	0.004	0.0
Cardiovascular disease (%)	137 (30.2)	35 (24.8)	102 (32.6)	0.119	0.0
Chronic pulmonary disease (%)	56 (12.3)	14 (9.9)	42 (13.4)	0.372	0.0
Malignant disease (%)	54 (11.9)	9 (6.4)	45 (14.4)	0.023	0.0
eGFR < 30 mL/min (%)	17 (3.7)	4 (2.8)	13 (4.2)	0.677	0.0
Obesity (%)	103 (22.7)	21 (14.9)	82 (26.2)	0.011	0.0
**Outcomes**					
Unfavorable outcome = yes (%)	92 (20.3)	0 (0.0)	92 (29.4)	<0.001	0.0
No ICU transfer (%)	380 (83.7)	141 (100.0)	239 (76.4)		
ICU transfer (%)	74 (16.3)	0 (0.0)	74 (23.6)		
Without ventilation (%)	16 (3.5)	0 (0.0)	16 (5.1)		
With non-invasive ventilation (%)	8 (1.8)	0 (0.0)	8 (2.6)		
With mechanical ventilation (%)	47 (10.4)	0 (0.0)	47 (15)		
With ECMO (%)	3 (0.7)	0 (0.0)	3 (1.0)		
Death (%)	43 (9.5)	0 (0.0)	43 (13.7)	<0.001	0.0

n = number of patients, p-value comparing groups, NA = data not available, IQR = interquartile range, GCS = Glasgow coma scale, NLR = neutrophile-to-lymphocyte ratio, PLR = platelet-to-lymphocyte ratio, CRP = C-reactive protein, eGFR = estimated glomerular filtration rate, ICU = intensive care unit, ECMO = extracorporeal membrane oxygenation.

**Table 2:** Laboratory results of COVID-19 patients among survivors and non-survivors (n = 454).

Variable	Overall	Survivor	Non-Survivor	*p*-Value	NA (%)
n (%)	454	411 (90.5)	43 (9.5)		
Gender				0.326	0.0
Male = yes (%)	291 (64.1)	260 (63.6)	31 (72.1)		
Female = yes (%)	163 (35.9)	151 (36.4)	12 (27.9)		
Severe = yes (%)	313 (68.9)	270 (65.7)	43 (100.0)	<0.001	1.3
NLR (median (IQR))	5.3 (3.3, 8.5)	5.0 (3.2, 8)	8.2 (6.6, 12.3)	<0.001	1.3
PLR (median (IQR))	222.9 (152.3, 334.3)	215.5 (148.9, 331.0)	268.3 (203.3, 425.0)	0.008	1.3
CRP (mg/L) (median (IQR))	65.3 (29.2, 120.0)	60.8 (27.6, 114.0)	102.0 (65.9, 150.8)	0.001	0.7

n = number of patients, NA = data not available, IQR = interquartile range, NLR = neutrophile-to-lymphocyte ratio, PLR = platelet-to-lymphocyte ratio, CRP = C-reactive protein.

**Table 3:** Proportional odds logistic regression for disease severity and binary logistic regression analysis for unfavorable outcome and mortality.

Laboratory Value	Outcome of Interest	Odds Ratio	95% CI	*p*-Value
**log (NLR)**				
	Disease severity	2.56	1.97–3.32	<0.0001
Age		1.02	1.00–1.03	0.014
Male		1.42	0.99–2.05	0.059
Comorbidities		2.09	1.43–3.06	0.0002
	Unfavorable outcome	2.04	1.46–2.91	<0.0001
Age		1.02	1.00–1.04	0.05
Male		1.88	1.09–3.34	0.027
Comorbidities		2.24	1.27–4.06	0.006
	Mortality	1.82	1.14–2.95	0.013
Age		1.09	1.05–1.13	<0.0001
Male		2.42	1.12–5.65	0.031
Comorbidities		1.13	0.52–2.62	0.76
**log (PLR)**				
	Disease severity	1.26	0.95–1.68	0.11
Age		1.02	1.01–1.04	0.0002
Male		1.69	1.18–2.41	0.004
Comorbidities		1.94	1.33–2.83	0.0006
	Unfavorable outcome	1.16	0.79–1.72	0.46
Age		1.02	1.01–1.04	0.009
Male		2.12	1.25–3.72	0.007
Comorbidities		2.14	1.23–3.85	0.008
	Mortality	1.37	0.79–2.46	0.27
Age		1.09	1.06–1.13	<0.0001
Male		2.59	1.21–6.00	0.018
Comorbidities		1.15	0.53–2.65	0.73
**log (neutrophil granulocytes)**				
	Disease severity	3.52	2.48–5.00	<0.0001
Age		1.02	1.01–1.04	<0.0001
Male		1.48	1.03–2.14	0.034
Comorbidities		1.78	1.22–2.61	0.003
	Unfavorable outcome	3.65	2.20–6.26	<0.0001
Age		1.03	1.01–1.05	0.004
Male		1.87	1.08–3.33	0.029
Comorbidities		1.92	1.09–3.50	0.027
	Mortality	1.98	0.99–4.08	0.057
Age		1.09	1.06–1.13	<0.0001
Male		2.45	1.14–5.69	0.028
Comorbidities		1.05	0.48–2.44	0.9
**log (lymphocytes)**				
	Disease severity	0.63	0.45–0.88	0.006
Age		1.02	1.01–1.03	0.001
Male		1.66	1.16–2.38	0.006
Comorbidities		2.06	1.41–3.01	0.0002
	Unfavorable outcome	0.83	0.54–1.27	0.4
Age		1.02	1.01–1.04	0.012
Male		2.11	1.24–3.70	0.007
Comorbidities		2.18	1.25–3.92	0.007
	Mortality	0.6	0.32–1.10	0.11
Age		1.09	1.05–1.12	<0.0001
Male		2.53	1.18–5.87	0.022
Comorbidities		1.18	0.54–2.72	0.69
**log (platelets)**				
	Disease severity	0.84	0.59–1.20	0.35
Age		1.02	1.01–1.04	<0.0001
Male		1.72	1.20–2.46	0.003
Comorbidities		1.92	1.32–2.80	0.0007
	Unfavorable outcome	1	0.60–1.78	0.99
Age		1.03	1.01–1.04	0.005
Male		2.06	1.22–3.59	0.008
Comorbidities		2.14	1.23–3.85	0.008
	Mortality	0.83	0.41–1.86	0.63
Age		1.09	1.06–1.13	<0.0001
Male		2.37	1.13–5.29	0.27
Comorbidities		1.16	0.54–2.67	0.71
**CRP**				
	Disease severity	1.01	1.01–1.01	<0.0001
Age		1.02	1.01–1.03	0.0009
Male		1.35	0.94–1.95	0.11
Comorbidities		1.81	1.23–2.65	0.002
	Unfavorable outcome	1.01	1.01–1.01	<0.0001
Age		1.02	1.01–1.04	0.009
Male		1.68	0.97–2.99	0.067
Comorbidities		2	1.14–3.66	0.19
	Mortality	1	1.00–1.01	0.042
Age		1.09	1.06–1.13	<0.0001
Male		2.15	1.01–4.86	0.054
Comorbidities		1.11	0.52–2.56	0.79

95% CI = confidence interval, NLR = neutrophil-to-lymphocyte ratio, PLR = platelet-to-lymphocyte ratio, CRP = C-reactive protein.

## Data Availability

Additional data are available upon reasonable request.

## Supplementary Materials

The following supporting information can be downloaded at https://www.mdpi.com/article/10.3390/diagnostics12051109/s1, Table S1: Definitions of clinical characteristics collected from patients hospitalized due to COVID-19. Figure S1: Individual slopes of summary measure analysis.
