# Supplementary material for: Neutrophile-to-Lymphocyte Ratio (NLR) Identifies Patients with Coronavirus Infectious Disease 2019 (COVID-19) at High Risk for Deterioration and Mortality—A Retrospective, Monocentric Cohort Study
Source: Diagnostics (Basel). 2022 Apr 28;12(5):1109. doi: 10.3390/diagnostics12051109 (PMC9139590; doi:10.3390/diagnostics12051109)

Table S1. Definitions of clinical characteristics collected from patients hospitalized due to COVID-19.

Variable	Definition	Unit
**Demographics**		
Gender	male or female	
Age		years
**Symptoms**	indicated as reported by the patient upon admission	
Time between onset of symptoms and hospitalization		days
Cough		
Fever	body temperature >38°C	°C
Headache		
Chest pain		
Dyspnea		
Myalgia, arthralgia, malaise		
Nasal congestion		
Gastrointestinal symptoms		
Sore throat		
Anosmia		
**Vital signs**	as documented upon hospital admission	
Systolic blood pressure		mmHg
Diastolic blood pressure		mmHg
Heart rate		/min.
Oxygen saturation without supplemental oxygen	oxygen saturation under room air without administration of supplemental oxygen	%
Respiratory rate		/min.
Temperature	body temperature measured by ear thermometer	°C
GCS	Glasgow coma scale indicating patients' consciousness, calculated from sum of the following points:<br>ocular response (1 = does not open eyes, 2 = opens eyes in response to pain, 3 = opens eyes in response to voice, 4 = opens eyes spontaneously)<br>oral response (1 = makes no sounds, 2 = makes sounds, 3 = words, 4 = confused, disoriented, 5 = oriented, converses normally)<br>motoric response (1 = makes no movements, 2 = extension to painful stimuli, 3 = abnormal flexion to painful stimuli,4 = flexion / withdrawal to painful stimuli, 5 = localizes to painful stimuli, 6 = obeys commands)	
**Comorbidities**	as documented in patients` personal history or diagnosed during hospital stay	
Arterial hypertension	systolic blood pressure ≥140 mmHg or a diastolic blood pressure ≥90 mmHg	
Diabetes mellitus	Hemoglobin A1c (HbA1c) ≥6,5% or fasting glucose ≥7.0 mmol/l or oral glucose tolerance test ≥11.1 mmol/l	
Cardiovascular disease	presence of coronary artery disease, occlusive peripheral artery disease, cerebrovascular disease (transient ischemic attack or stroke) and chronic heart failure	
Chronic pulmonary disease	Chronic obstructive pulmonary disease (COPD), pulmonary fibrosis, obstructive sleep apnea syndrome (OSAS), bronchial asthma	
Malignant disease	any current or preexisting hematologic or solid malignancy	
eGFR <30 ml/min	estimated glomerular filtration rate <30 ml/min./1.73 m^2 according to chronic kidney disease epidemiology collaboration (CKD-EPI) formula	
Obesity	BMI (body mass index) >30 kg/m^2	
**Laboratory results**	indicated as measured upon hospital admission	
NLR	Neutrophil-to-lymphocyte ratio	
PLR	Platelet-to-lymphocyte ratio	
Neutrophil granulocytes		10^9 cells/l
Lymphocytes		10^9 cells/l
Platelets		10^9 cells/l
CRP	C-reactive protein	mg/l

**Outcomes**	indicated as documented in medical report, worst classification reached during hospital stay
Disease severity	disease severity classification of COVID-19 according to World Health Organization (WHO) [8]:
- **Mild disease:**	uncomplicated illness:<br>Patients with uncomplicated upper respiratory tract viral infection, may have non-specific symptoms such as fever, cough, sore throat, nasal congestion, malaise, headache, muscle pain or malaise. The elderly and immunosuppressed may present with atypical symptoms. These patients do not have any signs of dehydration, sepsis, or shortness of breath.
- **Moderate disease:**	Mild pneumonia<br>Patients with pneumonia and no signs of severe pneumonia. (No need for supplemental oxygen)
- **Severe disease:**	Severe pneumonia<br>Patients with fever or suspected respiratory infection, plus one of the following: respiratory rate >30 breaths/min, severe respiratory distress, or peripheral capillary oxygen saturation (SpO2) <90% on room air.
- **Critically ill:**	Acute respiratory distress syndrome (ARDS):<br>Onset: new or worsening respiratory symptoms within one week of known clinical insult.<br>Chest imaging (radiograph, (computed tomography (CT) scan) bilateral opacities, not fully explained by effusions, lobar or lung collapse, or nodules.<br>Origin of oedema: respiratory failure not fully explained by cardiac failure or fluid overload.<br>- Mild ARDS: 200 mmHg < partial pressure of oxygen in blood (PaO2)/ inspired fraction of oxygen (FiO2) ≤300 mmHg (with positive endexpiratory pressure (PEEP) or continuous positive airway pressure (CPAP) ≥5 cmH2O,7 or non-ventilated)<br>- Moderate ARDS: 100 mmHg < PaO2/FiO2 ≤200 mmHg with PEEP ≥5 cmH2O,7 or non-ventilated)<br>- Severe ARDS: PaO2/FiO2 ≤100 mmHg with PEEP ≥5 cmH2O,7 or non-ventilated8)<br>• When PaO2 was not available, SpO2/FiO2 ≤315 suggests ARDS (including in non-ventilated patients)
Unfavorable outcome	Composite outcome of need for non-invasive ventilation (NIV), mechanical ventilation, extracorporeal membrane oxygenation (ECMO) or death
Death/Mortality	all-cause in-hospital mortality

Figure S1. Individual slopes of summary measure analysis (slope of NLR measurement of 244 survivors and 29 non-survivors): red dot showing each slope of survived patient and blue dot of non-survived patient, black dots representing the median slope in each group. Value of median slope is shown below the figure.

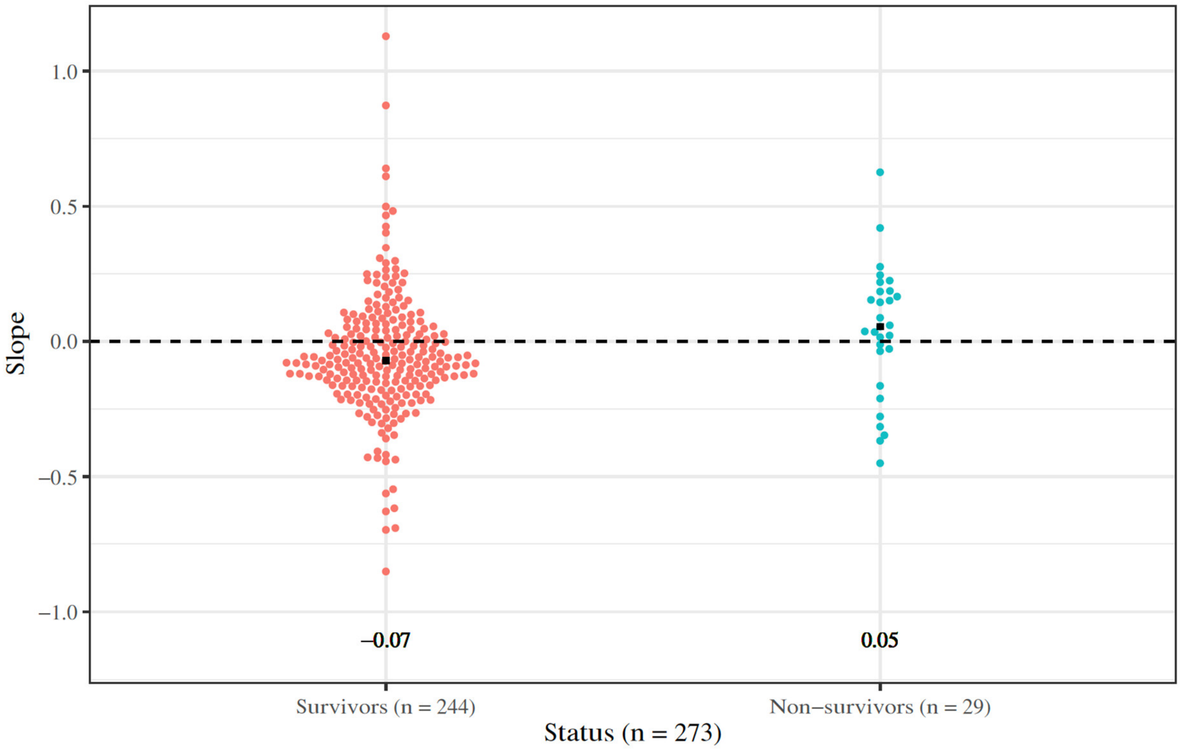

Supplement: Supplementary file 1 [file diagnostics-12-01109-s001.zip › diagnostics-1673289-supplementary.pdf]
